# Different treatments for acute myocardial infarction patients via outpatient clinics and emergency department

**DOI:** 10.1097/MD.0000000000013883

**Published:** 2019-01-11

**Authors:** Ching-Wen Chien, Cheng-Hua Wang, Zi-hao Chao, Song-Kong Huang, Pei-En Chen, Tao-Hsin Tung

**Affiliations:** a Institute for Hospital Management, Tsing Hua University, Shenzhen Campus, China; b Institute of Hospital and Health Care Administration, National Yang-Ming University, Taipei; c Faculty of Public Health, College of Medicine, Fu Jen Catholic University, New Taipei; d Association of Health Industry Management and Development; e Department of Medical Research and Education, Cheng Hsin General Hospital, Taipei, Taiwan.

**Keywords:** acute myocardial infarction, hierarchical logistic regression modeling, weekend effect

## Abstract

To investigate relevant factors and patients with acute myocardial infarction (AMI) were admitted during between weekdays and weekends period.

Retrospective population-based study setting: from the 2005 population-based national health insurance underwriting database of millions of people, random sampling (National Health Insurance Research Database [NHIRD]-Longitudinal Health Insurance Database [LHID] 2005).

In 2000 to 2009 data of NHIRD, subjects presented with first episode AMI who had received the thrombolytic therapy (TPA), or percutaneous coronary artery intervention (PTCA) or coronary artery bypass graft (CABG) during between weekdays and weekends period.

From 2000 to 2009 among patients with first AMI total of 2007 people, the weekday group 1453 people, the weekend group 554. The total mortality within 1 year showed 33.53%, the first-day mortality occupied 8.07% in 1 year of total mortality, increased mortality after admission 3 months later. Cox regression analysis showed that AMI has presented significant risk of death, there are 4 items: weekends, age, Charlson comorbidity index (CCI), thrombolytic therapy; in the other variables including emergency, hospital level, hospital ownership, and urban-rural gap are not significant differences. Further using hierarchical logistic regression analysis for Stratification of AMI mortality risk, it has significant that showed the hospital level, age, CCI, thrombolytic therapy; but emergency, PTCA and 3 CABG treatment are not significant differences.

It was approved by the hierarchical logistic regression analysis after stratified correction that the present study will have a direct impact on weekdays and weekends death in the hospital level. It will also affect the individual level.

**Key Points**

- On weekdays, hospitals generally allocate sufficient human resources to emergency departments to serve the higher patient load.
- The weekend effect may exist and reduce the overall medical service quality.
- Rapid hospital admission and treatment is key to ensuring a favorable prognosis for patients with acute myocardial infarction.
- This study adopted hierarchical logistic regression analysis for mortality rate stratification, revealing that hospital-level factors could directly affect mortality risks for both weekday and weekend admissions and influence patient-level factors.
- The adopted Cox regression analysis and hierarchical logistic regression analysis generated conflicting results, rendering the study unable to confirm the existence of the weekend effect.

## 1 Introduction

On weekdays, hospitals generally allocate sufficient human resources to emergency departments (ED) to serve the higher patient load. However, on holidays or weekends, hospitals tend to reduce the number of health professionals in ED or place less-experienced health professionals on duty, thus rendering weekend ED admissions relatively riskier than weekday ones. Therefore, the weekend effect may exist and reduce the overall medical service quality. In Taiwan, ED overcrowding has caused unequal medical resource allocation, crowding-out effects on medical resources, work and stress overload, unsatisfactory weekend medical service quality, and increased mortality risk, all of which require intervention.

Acute myocardial infarction (AMI) is a severe type of heart disease, leading to a first episode mortality rate of 44% to 55%. The longer the delay is in emergency treatment, the higher the mortality rate is. The American Heart Association reported that the mortality rate of patients with AMI is 6% within 6 hours of onset, 7% within 8 hours of onset, 8% within 12 hours of onset, and 16% for 12 hours or more after onset.^[1]^ Of patients with AMI who are not hospitalized within several hours of symptom onset, two-thirds die. Delayed treatment lead to mortality (mortality rate of 80%). Thus, rapid hospital admission and treatment is key to ensuring a favorable prognosis for patients with AMI.

Because patients with AMI experience a sudden onset of symptoms and require immediate treatment, admission method (emergency or outpatient) may affect their health and prognosis. This study examined the medical conditions of patients with AMI who were admitted on weekdays or weekends through various admission methods and who received various medical treatments in hospitals at different locations and of differing levels. The purpose of this study was to compare the differences in medical outcome and prognosis between patients with AMI admitted on weekdays and weekends, and to investigate the effects of the rural–urban development divide and hospital levels on these differences.

## 2 Materials and methods

### 2.1 Database

**Figure 1:**
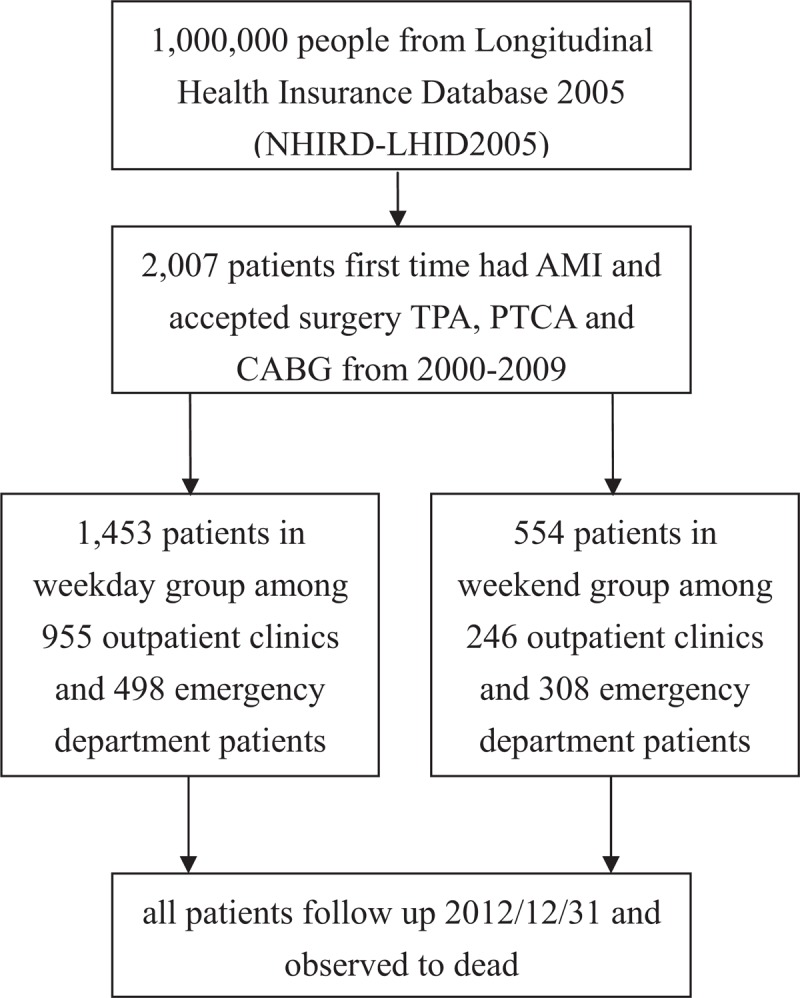
Flow chart of selection of the study population.

This retrospective study used the original claims data of 1 million National Health Insurance (NHI) beneficiaries who were enrolled in 2005 and were randomly sampled from the National Health Insurance Research Database (NHIRD) into the Longitudinal Health Insurance Database 2005 (LHID2005). This study analyzed the samples of patients who experienced their first episode of AMI (ICD-9 410.x1) in 2000 to 2009, excluding those who were not admitted for the first time through inpatient admission, did not specify their sex, or were aged less than 18 years. In addition this study analyzed these samples by identifying outcome differences between those admitted on weekdays and weekends after they received tissue plasminogen activator (TPA) treatment, percutaneous transluminal coronary angioplasty (PTCA), or a coronary artery bypass graft (CABG). The adopted independent variables were admission method and treatment type, and the adopted dependent variable was 1-year mortality rates after symptom onset. The patients were followed up until withdrawal from the NHI, death, or December 31, 2010. Figure 1 lists the sampling procedures. Because the data source is in the public domain and anonymized, the present study has been exempted from review by the Institutional Review Board of Taipei Veterans General Hospital (IRB-TPEVGH No: 2015–11–001BC).

### 2.2 Study variables

The adopted variables are detailed as follows:

1. Independent variables
  Admission time (weekend or weekday (ie, workday)): by referring to the 2000 to 2009 Work Calendars released by the Directorate- General of Personnel Administration of Taiwan's Executive Yuan, this study divided hospital emergency admission into 2 periods: weekends: Saturdays, Sundays, and holidays, including national holidays, festival days, memorial days, and deferred holidays; weekdays: days other than weekends and holidays.
  Admission method (emergency or outpatient): emergency admission was defined as a medical admission that incurred emergency diagnostic fees or hospitalization fees for staying in the ED for further observation. The emergency diagnostic codes used in the database were 01015C, 00201, 00202A, 00203A, and 00204A. The codes for ED stays were 03018A and 03019B.
  Treatment type:
  (1) TPA Taiwan NIH code: metalyse (K000714229), actilyse (K000743248), streptase (B021919289), and urokinase (A046226205/ AC46226205) (B021379265/ BC21379265);
  (2) PTCA Taiwan NIH code: 33076A–33078A, 33076B–33078B, 97511K, 97512A, 97513B, 97516K, 97517A, 97518B, 97521K, 97522A, and 97523B;
  (3) CABG Taiwan NIH code: 68023A–68025A, 68023B–68025B, 97901K, 97902A, 97903B, 97906K, 97907A, 97908B, 97911K, 97912A, 97913B, 97916K, 97917A, and 97918B;
  (4) CABG Taiwan NIH code: 361.X and 362.
  Control variables
  Hospital levels: according to the registry for contracted medical facilities and registration files from the NHIRD, the contracted category (HOSP_CONT_TYPE) of hospitals was divided into 3 levels: medical centers (including medical center candidates; code 01), regional hospitals (including regional hospital candidates; code 02), and district hospitals (including district teaching hospitals; code 03).
  Hospital ownership: The NHIRD groups hospitals into the following 3 types depending on ownership:
  (1) public hospitals: hospitals subordinate to the Department of Health (DOH; now the Ministry of Health and Welfare) and municipal hospitals (ownership code 01), county and city hospitals (ownership code 02), hospitals affiliated with public medical schools (ownership code 04), military hospitals (ownership code 05), veteran hospitals (ownership code 06), and hospitals affiliated with public enterprises (ownership code 07);
  (2) hospitals affiliated with nonprofit organizations: nonprofit proprietary hospitals (ownership code 11) and hospitals affiliated with nonprofit proprietary religious organizations (ownership code 12); and
  (3) private hospitals: hospitals affiliated with medical schools (ownership code 13), hospitals affiliated with other nonprofit proprietary organizations (ownership code 14), and private hospitals (ownership code 15).
  Degree of urbanization: The statistics database of the Bureau of Labor Insurance defines an area as being urban (codes 1 and 2), suburban (codes 3 and 4), or rural (code 5–7) depending on degree of urbanization.^[2]^
  Charlson Comorbidity Index (CCI): The risk adjustment for the samples’ disease severity was conducted using this classifying method of prognostic comorbidity employed by the previous definitions.^[3,4]^
2. Dependent variables

Death status: Death was determined according to patient status codes (ie, TRAN_CODE) recorded in inpatient claims data (DD file; code 4: death, code 5: discharge against advice, and code A: critical discharge against advice) and the insurance status codes (ie, ID_OUT_TYPE) recorded in the NHI underwriting database (code 1: insurance withdrawal; code 5: insurance suspension).

Mortality risk: This study examined mortality risk of the patients admitted in 2000 to 2009 and followed up within a year after discharge for the following periods: 1, 2, 7, 14, 30, 90, and 180 days and 1 year.

### 2.3 Patient and public involvement

This retrospective population-based study is based on NHI Database. Study subjects were not involved in the recruitment or conduct.

The study result did not be disseminated directly to patients, although the findings informed quality improvement initiatives. The study also did not include patient advisors.

### 2.4 Statistical analysis

This study used SAS version 9.3 for statistical analysis and conducted the chi-squared test and independent sample *t* test for univariate correlation analysis. Inferential statistics were employed using the Kaplan–Meier estimator (for analyzing patient survival between the weekend and weekday samples), a Cox regression analysis (for multivariate analysis), and a hierarchical logistic regression model to divide hospital and patient levels and reconfirm death status (*P* <.05).

## 3 Results

As Table 1 shows, from 2000 to 2009 among patients with first AMI total of 2007 people, the weekday group 1453 people, the weekend group (n = 554), ER (n = 806), OPD (n = 1201), men (n = 1370), female (n = 637), average age 69.5 ± 11.6 years. A significant difference of admission was observed between the weekday and weekend samples (*P* <.001).

**Table 1:**
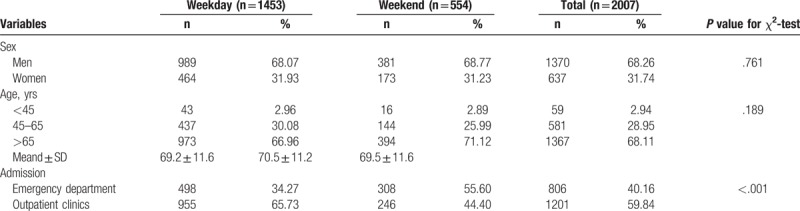
The baseline characteristics of acute myocardial infaraction patients admitted between weekday and weekend (n = 1370).

Table 2 reveals that 53 patients received TPA treatment, accounting for 2.6% of all patients and indicating a significant correlation between TPA treatment and admission time (*P* = .009). A total of 138 patients received both PTCA and CABG treatment, accounting for 6.8% of all patients and suggesting a significant correlation between these treatments and admission time (*P* = .035). In addition, for all admission times, more than 80% of the patients received PTCA treatment and more than 20% of them received CABG treatment. Moreover, on weekends, more than 70% of the patients received PTCA treatment and more than 20% of them received CABG treatment. No significant difference in average hospitalization fees or average length of stay was observed between weekdays and weekends.

**Table 2:**
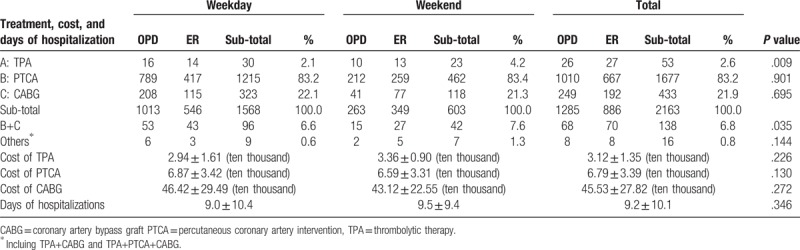
The treatment distribution of acute myocardial infaraction patients between weekday and weekend.

Table 3 shows that 1001 patients were admitted to medical centers (49.88% of all patients), 937 were admitted to regional hospitals (46.69%), and 69 were admitted to district hospitals (3.44%). On weekdays, a total of 1453 patients were admitted (693 to medical centers (47.69%), 715 to regional hospitals (49.21%), and 45 to district hospitals (3.10%)), with the results attaining significant difference (*P* = .019). On weekends, a total of 554 patients were admitted (308 to medical centers (55.06%), 222 to regional hospitals (40.07%), and 24 to district hospitals (4.33%)), revealing a significant difference (*P* = .004). Thus, no significant difference in hospital ownership or degree of urbanization was observed between weekends and weekdays.

**Table 3:**
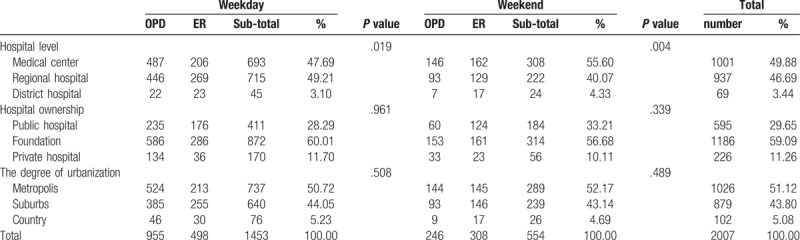
The medical sources for acute myocardial infaraction patients via outpatient clinics and emergency department admitted between weekday and weekend.

**Figure 2:**
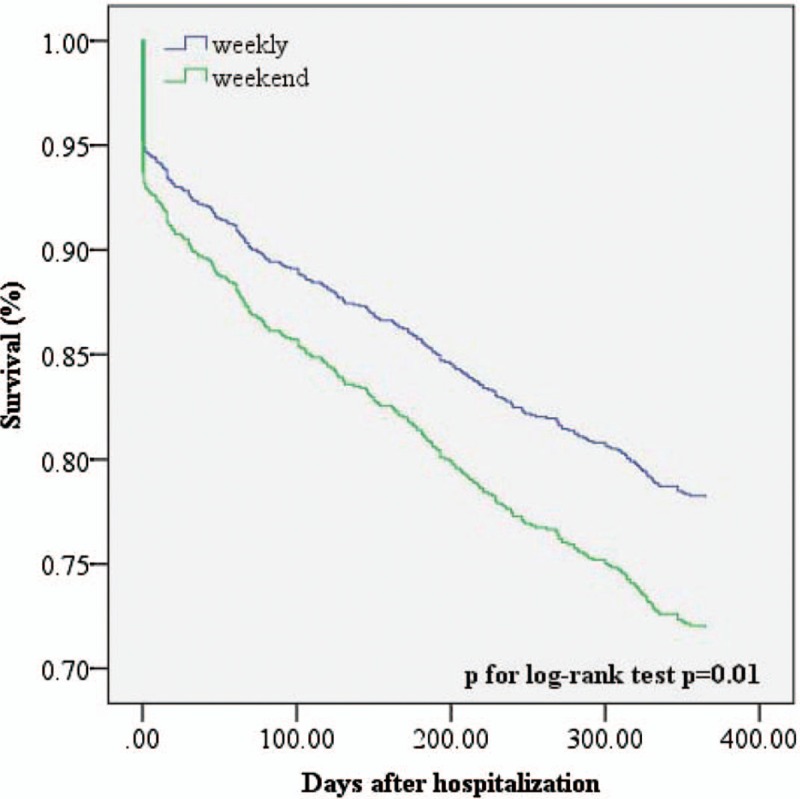
The survival rate of acute myocardial infaraction patients between weekday and weekend.

Figure 2 shows that the survival rate of the patients admitted on weekends was lower than that of patients admitted on weekdays (*P* for log-rank test = .01). The Cox regression results in Table 4 indicate that the mortality risk of the weekend patients was 1.363 times that of the weekday patients (95% confidence interval [CI]: 1.076–1.726), that higher ages were associated with higher mortality risks (HR = 1.035, 95% CI: 1.022–1.047), and that the mortality risk of the patients who received TPA treatment was 3.208 times that of those who did not receive such treatment (95% CI: 1.883–5.466).

**Table 4:**
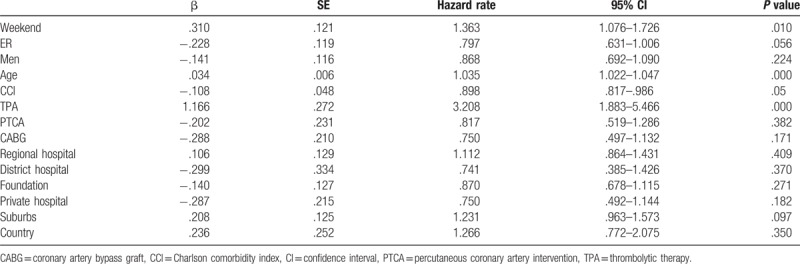
Cox regression of risk of mortality among acute myocardial infaraction patients.

Hierarchical logistic regression modeling was conducted to examine the effects of hospital and patient levels on mortality risk. After analyzing the impact of hospital levels on mortality risk, this study substituted the resulting estimated intercepts into the hierarchical regression model for patient-level analysis. Table 5 indicates that a significant difference was observed between hospital levels and mortality risk (estimate: −2.2964; *P* = .0072; 95% CI: 3.9385 to −0.6543), that no significant differences were observed after hierarchical logistic regression modeling was conducted on the effects of admission time, that higher ages were associated with higher mortality risks (estimate: 0.041; *P* <.0001; 95% CI: 0.02711–0.05488), that higher CCI values were associated with higher mortality risks (estimate: −0.1192; *P* = .0311; 95% CI: −0.2275 to −0.01086), and that reduced mortality risks were observed in patients who did not receive TPA treatment (estimate: −1.737; *P* = .0002; 95% CI: −2.6521 to −0.822), suggesting higher mortality risks for those who received TPA treatment. In addition, no significant differences were observed between mortality risk and the following variables: adoption of PTCA and CABG treatment, emergency admission, and sex.

**Table 5:**
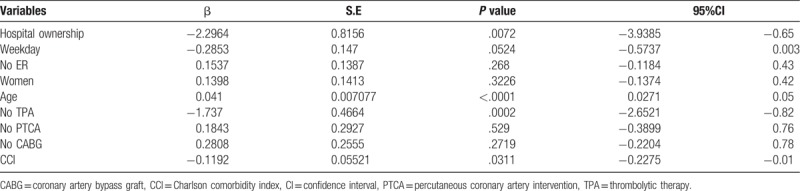
Hierarchical logistic regression modeling for the effects of hospital and patient levels on mortality risk.

## 4 Discussion

### 4.1 Clinical implication

This study compared the mortality risk of patients with first AMI onset in 2000 to 2009 who were admitted on weekends with the mortality risk of those admitted on weekdays. The 1-day and 2-week mortality rates of the weekend patients were found to be higher than those of the weekday patients, suggesting that the weekend effect may have affected the mortality risk of the patients for 2 weeks after admission. The results of the Cox regression analysis showed that the mortality risk of the weekend patients was 1.363 times that of the weekday patients, similar to the results of previous studies.^[5–8]^ Li (2012) empirically demonstrated that the 7-, 30-, and 180-day mortality rates of patients admitted on weekends or Chinese New Year holidays were higher than those of patients admitted on weekdays and that this negative weekend effect mostly occurred in nonmedical centers rather than in medical centers. Thus, the hierarchical logistic regression modeling results generated in the present study revealed that hospital levels not only directly affected the mortality rate of patients admitted on weekends and weekdays but also influenced patient levels.^[9]^

To examine the effect of admission method, this study conducted a univariate correlation analysis, showing that the 1-day and 2-week mortality rates of the weekend patients were higher than those of the weekday ones. Therefore, the weekend effect may have affected the 2-week mortality risk of weekend samples admitted through the EDs. This can be attributed to the fact that the patients with less severe symptoms generally visited outpatient departments, whereas those with more severe symptoms were admitted through EDs. In addition, this study conducted a Cox logistic regression analysis to calculate the hazard ratio (HR) of the weekend samples (HR: 0.797; 95% CI: 0.631–1.006), and a performed hierarchical logistic regression analysis on the same samples (*P* = .0524; 95% CI: −0.5737 to 0.003). The inferential statistical results indicated that no significant differences were observed in mortality risk between outpatient and emergency admission, rendering the study unable to confirm the existence of the weekend effect in the context of weekend emergency admissions. Sharp et al (2013) reported that, in the context of emergency admissions, the mortality risk of adults admitted on weekends through EDs was significantly higher than that of adults admitted on weekdays (odds ratio [OR]: 1.073; 95% CI: 1.061–1.084). After controlling for patient characteristics, the following results were obtained: an adjusted OR (AOR) of 1.026 (95% CI, 1.005–1.048). Sharp et al (2013) did not identify the existence of the weekend effect even after adjusting patient income, insurance status, hospital ownership, ED volume, and hospital teaching status.^[10]^

The results of the Cox logistic regression analysis and hierarchical logistic regression analysis indicated that the mortality risk of patients who received TPA treatment was 3.12 times that of patients who did not receive the treatment, suggesting an increased mortality risk among the specific group of patients (average age: 71.90 ± 13.14; average CCI: 1.83 ± 1.02). Moreover, such increased risk can be attributed to several factors causing admission delays, including high disease severity, high ED volume, long referral time, and high cardiac catheterization room volume. The 2013 ACCF/AHA Guideline for the Management of ST-Elevation Myocardial Infarction, which emphasizes advances in reperfusion therapy, indicates that, in the absence of contraindications, fibrinolytic therapy (a TPA treatment) should be administered to patients when the anticipated transferal time exceeds 120 minutes.^[11]^ Vora et al (2015) reported that patients treated with fibrinolytic therapy did not have significant mortality difference compared with those treated with primary percutaneous coronary intervention (pPCI) (3.7% vs 3.9%; AOR: 1.13; 95% CI: 0.94–1.36), but that they had a higher bleeding risk (10.7% vs 9.5%; AOR: 1.17; 95% CI: 1.02–1.33) and that, for patients unlikely to receive timely pPCI treatment, pretransfer fibrinolysis and an early transfer for angiography may be a suitable reperfusion option when the potential benefits of timely reperfusion outweigh bleeding risk.^[12]^ Furthermore, most countries have recognized pPCI as an effective method of clearing a blocked coronary artery for patients with AMI. It has a 90% surgical success rate and reduces 24-hour mortality risk and diminishes 30-day reinfarction rate. However, more than one-third of reinfarctions occur within a year after PTCA treatment. Thus, to reduce further stenosis, patients can receive a coronary stent implantation after balloon angioplasty. The stenosis incidence rate can be reduced to 15% to 20% by metal coronary stent implantation and to 5% by drug-eluting stent implantation.

This study observed that, for both weekdays and weekends, more than 80% of the patients received PTCA treatment and more than 20% of the samples received CABG treatment, and that a total of 138 patients received both PTCA and CABG treatment, accounting for 6.8% of all patients. Although the NHIRD did not provide disease severity indicators, this study considered patients who received 2 or more treatments as an indicator of high disease severity because these patients generally experienced more severe symptoms or riskier treatments than the other patients did. The Cox and hierarchical logistic regression results revealed no significant differences between PTCA and CABG treatment and mortality risk, which was similar to the results of other studies. Previous study suggested that the proportion of patients with AMI who received pPCI substantially increased annually, from 12.4% in 1996 to 54.7% in 2007.^[13]^

The mortality risk of the patients who received pPCI was affected by factors including income, insurance, aspirin intake, admission time (working hours or nonworking hours; weekdays or weekends), hospital ownership, ED volume, number of cardiology physicians, physician experience with pPCI, and door to balloon (D2B) time.^[10,14,15]^ Accordingly, a high-quality cardiology center equipped with a solid network connecting each department or division, comprehensive medical treatment planning, and experienced attending physicians can ensure no significant differences in mortality risk and provide effective pPCI treatments to patients regardless of admission time.^[16]^

Each year, more than 20% of the patients received CABG treatment, whereas 6.8% of the patients received pPCI followed by CABG (indicating riskier treatment conditions or higher disease severity). Mehta et al (2013) found that, after receiving pPCI with stent implantation, patients with ST-segment elevation myocardial infarction received CABG because of stenosis recurrence and vessel malformation or rupture, exhibiting mortality rates of 10% and 20% respectively. For patients requiring early CABG, monitoring TIMI 3 flow rate is a safe option to enable delivering a timely treatment for early ischemia.^[17]^

The present study observed that the mortality rates of the patients began to increase substantially 3 months after their first ED admission, the reasons of which may be similar to the findings of a study examining patients with AMI in New Jersey hospitals.^[18]^ In recent decades, inpatient mortality has decreased substantially, whereas the decrease in long-term mortality has been less noticeable, suggesting that the mortality rate increased after the patients were discharged, mainly because of high noncardiovascular mortality, especially from respiratory and renal diseases, septicemia, and cancer in senior patients.^[18]^Table 6 presents the different treatments for AMI patients in various populations.^[1,3–8,10,12,14–20]^ In addition to diagnostic criteria, this disparity is largely due to the different sources of AMI patients. From 1996 to 2006, the inpatient AMI mortality rate declined annually, a phenomenon similar to the decreasing AMI mortality rate reported by the Ministry of Health and Welfare in their statistics that was corrected by the 2000 to 2025 population-adjusted mortality rate estimated by the World Health Organization. The 2005 to 2008 AMI mortality rate determined in the LHID under the NHIRD showed a similar decreasing trend.

**Table 6:**
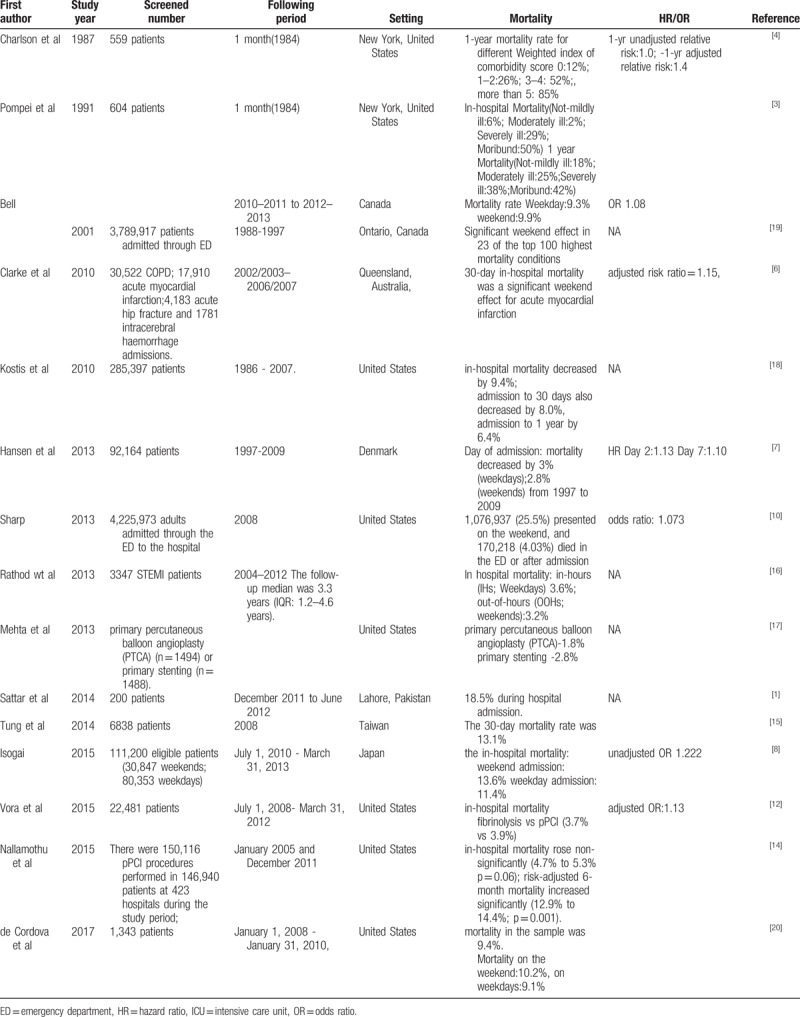
The different treatments for acute myocardial infarction patients in various populations.

For patients who experienced their first AMI episode, the present study found a 1-day mortality rate of 8.07%, a 2-week mortality rate of 10.47%. The mortality rate increased substantially 3 months after AMI onset and the trend continued; the 1-year total mortality rate reached 33.53% (67.63% for weekday patients and 32.37% for weekend patients). In addition, although the number of male patients far exceeded that of female patients and despite the male patients having an AMI incidence rate 2.15 times that of the female patients, no significant mortality rate differences were observed between the sexes in a modeling analysis. After analyzing the age variable through a regression analysis, this study found that older age was associated with higher mortality risks, which can be attributed to the fact that the patients aged >65 years accounted for 68.11% of all patients and that the patients who experienced CCI ≥1 accounted for 71.75% of all patients, conforming to past study results.^[18]^

### 4.2 Research limitations

First, this study determined death status according to the patient status codes (ie, TRAN_CODE) recorded in the inpatient claims data (DD; code 4: death, code 5: discharge against advice, and code A: critical discharge against advice) and the insurance status codes (ie, ID_OUT_TYPE) recorded in the NHI underwriting database (code 1: insurance withdrawal and code 5: insurance suspension). National death registry data are more accurate than these data but could not be obtained in this study. Therefore, although death status could have been overestimated, these codes generally represented patient death because of the compulsory nature of Taiwan's NHI program; thus they were accurate indicators of death. Second, this study did not distinguish the nature of holidays, the date of which might have fallen on weekdays or weekends, thereby affecting the study results. Third, this study did not distinguish admission method on Saturdays because some hospitals in Taiwan provide both emergency and outpatient services, which may have affected the results of identifying the weekend effect. Finally, this study did not include various variables not provided in the NHIRD, such as times (eg, working and nonworking hours, D2B time, and referral time), disease severity, number and quality of medical professionals on duty, physician service length and experience, ED service volume, ED overcrowding status, and cardiac catheterization room status, leading to possible bias in the analysis results.

## 5 Conclusion

When examining the weekend effect, previous studies have mainly employed single-level analysis and thus have been unable to identify differences between weekdays and weekends. However, this study adopted hierarchical logistic regression analysis for mortality rate stratification, revealing that hospital-level factors could directly affect mortality risks for both weekday and weekend admissions and influence patient-level factors. When examining the mortality risk of the patients admitted on weekdays and weekends, this study conducted a univariate analysis and found that the 1-day and 2-week morality rates of the weekend patients were higher than those of the weekday patients, indicating that the weekend effect may have affected the mortality rates of those admitted on weekends for the 2 weeks after they were admitted. Furthermore, the adopted Cox regression analysis and hierarchical logistic regression analysis generated conflicting results, rendering the study unable to confirm the existence of the weekend effect.

## Acknowledgment

This study was also supported by master thesis from the Cheng-Hua Wang, “A Comparison on Outcomes of Accepted Different Treatments for Acute Myocardial Infaraction Patients via Outpatient Clinics and Emergency Department Admitted between Weekday and Weekend”, Institute of Hospital and Health Care Administration, National Yang-Ming University, Taipei, Taiwan, July 2015.

## Author contributions

**Conceptualization:** Ching-Wen Chien, Cheng-Hua Wang.

**Data curation:** Pei-En Chen.

**Formal analysis:** Zi-hao Chao, Song-Kong Huang.

**Methodology:** Zi-hao Chao, Song-Kong Huang.

**Writing – original draft:** Cheng-Hua Wang.

**Writing – review & editing:** Ching-Wen Chien, Cheng-Hua Wang, Tao-Hsin Tung.
